# Transcriptomic Profiling and Gene Disruption Revealed that Two Genes Related to PUFAs/DHA Biosynthesis May be Essential for Cell Growth of *Aurantiochytrium* sp.

**DOI:** 10.3390/md16090310

**Published:** 2018-09-01

**Authors:** Yuanmei Liang, Ying Liu, Jie Tang, Jiong Ma, Jay J. Cheng, Maurycy Daroch

**Affiliations:** 1 School of Environment and Energy, Peking University Shenzhen Graduate School, Shenzhen 518055, China; lym@sz.pku.edu.cn (Y.L.); jiongm@gmail.com (J.M.); chengjy@pkusz.edu.cn (J.J.C.); m.daroch@pkusz.edu.cn (M.D.); 2 Guangdong Engineering Research Centre for Marine Algal Biotechnology, Shenzhen Key Laboratory of Marine Bioresource and Eco-environmental Science, College of Life Sciences and Oceanography, Shenzhen University, Shenzhen 518060, China; 3 School of Pharmacy and Bioengineering, Chengdu University, Chengdu 610106, China; tangjie@pkusz.edu.cn; 4 Department of Biological and Agricultural Engineering, North Carolina State University, Raleigh, NC 27695, USA

**Keywords:** thraustochytrid, *Aurantiochytrium*, transcriptome, very-long-chain (3R)-3-hydroxyacyl-CoA dehydratase, dehydrase/isomerase

## Abstract

*Aurantiochytrium* sp. PKU#SW7 is a thraustochytrid strain that was found to exhibit high potential for docosahexaenoic acid (DHA, C22:6n-3) production. In this work, the transcriptome of *Aurantiochytrium* sp. PKU#SW7 was analyzed for the study of genes involved in basic metabolic functions and especially in the mechanisms of DHA biosynthesis. Sequence annotation and functional analysis revealed that the strain contains components of fatty acid synthesis (FAS) and polyketide synthase (PKS) pathways. Fatty acid desaturases and elongases were identified as components of FAS pathway, whilst key components of PKS pathway were also found in the cDNA library. The relative contribution of the two pathways to the synthesis of DHA was unknown, as both pathways appeared to be lacking full complement of genes for standalone synthesis of DHA. Further analysis of two putative genes encoding the very-long-chain (3R)-3-hydroxyacyl-CoA dehydratase and dehydrase/isomerase involved in FAS and PKS pathways, respectively, revealed that under various salinity conditions, their relative expression levels changed corresponding to the variation of DHA content in *Aurantiochytrium* sp. Independent knock outs of these genes in *Aurantiochytrium* sp. resulted in poor cell growth, probably due to little or no intracellular DHA accumulation. Hence, it can be speculated that both genes are engaged in DHA biosynthesis and DHA in *Aurantiochytrium* sp. could be produced by jointed actions of both FAS and PKS systems.

## 1. Introduction

Thraustochytrids are algae-like marine protists, belonging to the class Labyrinthulomycetes, and were first described by Sparrow [1]. Thraustochytrids have been reported to play multiple important roles in marine ecosystem, and were potentially involved in different processes in marine food webs [2,3,4,5]. Moreover, it is worth emphasizing that thraustochytrids can accumulate a large amount of fatty acids. Especially, the high content of omega-3 polyunsaturated fatty acids (PUFAs) such as eicosapentaenoic acid (EPA) and docosahexenoic acid (DHA) recovered from thraustochytrids cells and oils gained much attention. Some previous studies have successfully applied thraustochytrids oil as a renewable PUFAs source for fish diets [6,7]. Particularly, DHA can be used as a nutraceutical due to its importance in human physical health, especially in maturing of the retinal cells and development of the brain [8,9,10,11]. Companies around the world have also developed thraustochytrids oil products for human consumption [12]. To attain a high yield of DHA from thraustochytrids, numerous researches have been conducted on optimizing the cultivation parameters, including culture medium nutrients, incubation temperature, salinity, pH, and dissolved oxygen level [13,14,15,16]. Metabolic engineering can show promise of further increasing the DHA productivity of thraustochytrids. Detailed understanding of DHA biosynthesis is therefore essential for efficient modification of the metabolism. Two distinct PUFAs biosynthesis pathways yielding DHA, namely, fatty acid synthesis (FAS) and polyketide synthesis (PKS) pathways have been observed in thraustochytrids [17,18]. The conventional FAS pathway comprised of a typical fatty acid synthesis metabolism followed by a series of desaturation and elongation reactions yielding long chain polyunsaturated DHA. Some of the key enzymes, including Δ4-desaturase, Δ5-desaturase, Δ5-elongase, Δ6-elongase and Δ12 desaturase, have been successfully identified in some thraustochytrid strains [17,19,20]. However, in *Schizochytrium* or *Aurantiochytrium* strains, the FAS pathway was mostly reported as incomplete [19]. The alternative PKS pathway was first reported in a *Schizochytrium* strain by Metz et al. [21]. Polyketides, as a diverse group of complex organic compounds, are synthesized through repetitive decarboxylative condensation of coenzyme A (CoA) for carbon chain extension [22]. Distinct from the FAS desaturase/elongase pathway, PUFAs are synthesized via a series of iterative processing, trans-cis isomerization and enoyl reductions with malonyl-CoA as the initial substrate in PKS pathway [21,23]. So far, the exact mechanism of PUFAs biosynthesis originating from the PKS pathway remains to be determined in detail.

In our previous study, a thraustochytrid strain, *Aurantiochytrium* sp. PKU#SW7 was isolated [24] and found to exhibit high potential for DHA production [16]. However, the DHA biosynthesis pathway in *Aurantiochytrium* sp. has not been fully elucidated yet [25] and to date there is no relevant genomic data available to resolve the issue of DHA synthesis mechanism in *Aurantiochytrium*. High-throughput RNA-sequencing (RNA-Seq) technique offers researchers an important tool to analyze the characteristics and function of transcriptomes, especially when genomic information is lacking [26]. A large number of previous researches have adopted RNA-Seq for the study of specific functional mechanism or acclimation of oleaginous microorganisms [25,27,28].

In this work, the transcriptome of *Aurantiochytrium* sp. PKU#SW7 was analyzed for the studying of genes involved in basic metabolic functions and specifically in the potential mechanisms of DHA biosynthesis. Two candidate genes potentially involved in FAS and PKS pathways were selected for further functional analysis through generation of individual gene knock-outs by homologous recombination. Results of the present study will provide insights into the molecular mechanism of DHA synthesis and become valuable sources to identify genetic manipulation strategies aiming for improved DHA production in the future.

## 2. Results and Discussion

### 2.1. De Novo Assembly and Functional Annotation of Aurantiochytrium Transcriptome

**Figure 1:**
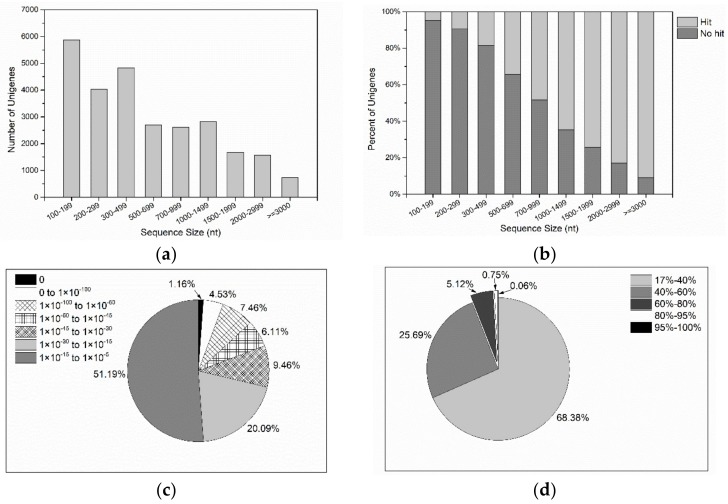
De novo assembly and functional annotation of *Aurantiochytrium* transcriptome. (**a**) Sequence length distribution of unigenes; (**b**) percent of successful (hit) and unsuccessful (no hit) annotated unigenes through functional annotation of *Aurantiochytrium* sp. PKU#SW7 trancriptome; (**c**) E value distribution of BLAST hit for the assembled unigenes with a cutoff of 1×10^−5^ in Nr database; (**d**) Similarity distribution of the top BLAST hits for the assembled unigenes with a cutoff of 1×10^−5^ in Nr database.

Sequencing of cDNA libraries prepared under DHA biosynthesis conditions [16] generated a total of 28,762,644 raw reads. After a series of filtering and assembling steps, a total of 26,845 unigenes were further generated, and their length varied from 150 to 9693 bp with an average of 767 bp and N50 value of 1372 bp (Figure 1a). 

Functional annotation of all the unigenes revealed that, a total of 10,070 unigenes were matched to the deposited protein with putative or unknown functions, accounting for 37.51% of the total assembled unigenes. Thereinto, 8794 (32.76%) unigenes exhibited a notable hit with known proteins in the Nr database (the NCBI nonredundant protein database). 74.10% of the unigenes over 1000 bp in length showed homologous matches, especially over 90% of the unigenes with length longer than 3000 bp matched to the Nr database. However, only 6.71% of the unigenes shorter than 300 bp showed matches (Figure 1b). The results revealed that longer sequences were more likely to match deposited proteins in the public database, which was corresponding to most previous transcriptome studies [29,30,31]. The E value distribution of the top hits revealed that only 19.26% of the aligned sequences showed striking homology to entries in the Nr database (*E* value < 1×10^−45^) (Figure 1c) and less than 1% of the sequences showed more than 80% similarity (Figure 1d). The low hit percentage in the present study may indicate that there is limited protein information of *Aurantiochytrium* and other related thraustochytrid species in Nr database and those reads with no considerable similarity with the known proteins might encode novel functions specific to thraustochytrids.

### 2.2. Function Classification and Pathway Analysis

**Figure 2:**
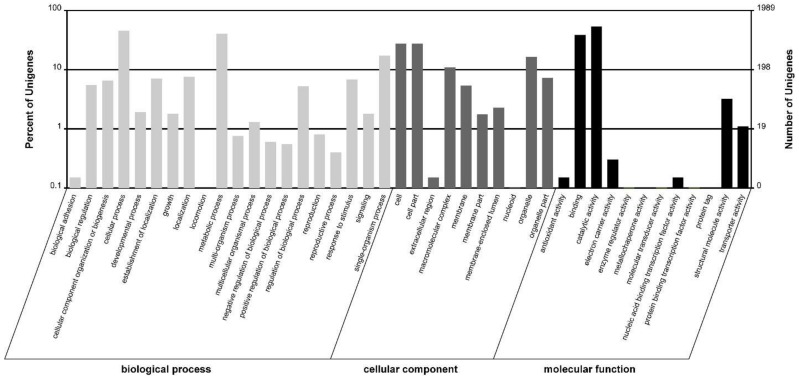
Functional categories of *Aurantiochytrium* sp. PKU#SW7 trancriptome based on GO annotation. A total of 1989 unigenes were assigned at least one GO term and divided into three categories and 42 subcategories.

As an international standardized gene functional classification system, GO (gene ontology) assignments were used to classify the functions of *Aurantiochytrium* transcripts based on the sequence similarity to protein sequences in the Nr annotation. Out of the 8794 Nr annotated unigenes, 1989 of them were assigned at least one GO term, and were categorized into 42 functional groups (Figure 2). Assignments to the biological process made up the majority (3023, 43.66%), followed by cellular component (1972, 28.48%), and molecular function (1929, 27.46%). Among the biological process group, most of the unigenes had potential functions related to cellular processes (904, 29.90%), metabolic processes (804, 26.60%), and single-organism processes (344, 11.38%). Under the cellular component group, cell (545, 27.64%) and cell part (545, 27.64%) were most highly represented, followed by organelle (326, 16.53%) and macromolecular complex (219, 11.11%). Catalytic activity (1060, 54.95%) and binding (763, 39.55%) made up the vast majority in the molecular function groups. As reported by Huang et al. [32], the binding proteins and catalytic activity proteins (enzymes) (77.55%) also composed the majority of *Schizochytrium* ESTs. The highly represented enzymes and binding proteins indicated that considerable enzymes within *Aurantiochytrium* may be involved in binding with substrate in DHA biosynthesis pathways [32]. 

**Figure 3:**
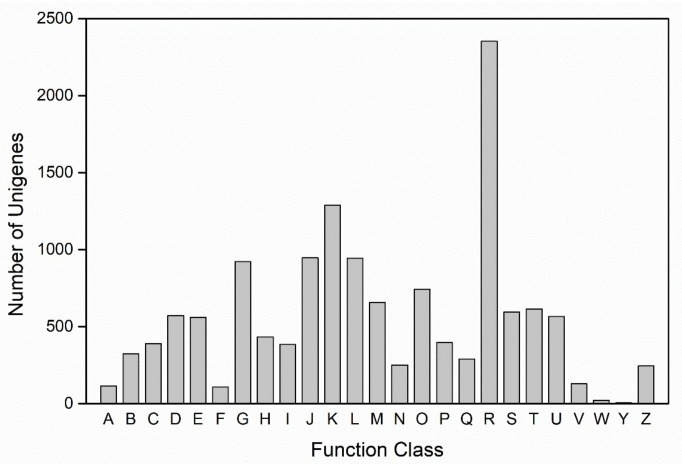
Cluster of orthologous groups (COG) classification. A total of 6042 unigenes were classified into 25 categories. A: RNA processing and modification; B: Chromatin structure and dynamics; C: Energy production and conversion; D: Cell cycle control, cell division, chromosome partitioning; E: Amino acid transport and metabolism; F: Nucleotide transport and metabolism; G: Carbohydrate transport and metabolism; H: Coenzyme transport and metabolism; I: Lipid transport and metabolism; J: Translation, ribosomal structure and biogenesis; K: Transcription; L: Replication, recombination and repair; M: Cell wall/membrane/envelope biogenesis; N: Cell motility; O: Posttranslational modification, protein turnover, chaperones; P: Inorganic ion transport and metabolism; Q: Secondary metabolites biosynthesis, transport and catabolism; R: General function prediction only; S: Function unknown; T: Signal transduction mechanisms; U: Intracellular trafficking, secretion, and vesicular transport; V: Defense mechanisms; W: Extracellular structures; Y: Nuclear structure; Z: Cytoskeleton.

The functions of unigenes were also classified according to the COG database (the Cluster of Orthologous Groups database), through which, a total of 6042 unigenes were classified into 25 categories. The cluster of general function prediction only (2354, 38.96%) represented the largest group, followed by transcription (1288, 21.32%). Unigenes assigned to extracellular structures and nuclear structure were the least represented (Figure 3).

The KEGG database (the Kyoto Encyclopedia of Genes and Genomes KEGG pathway database) was further applied for the identification of unigenes involved in metabolic pathways. A total of 6941 unigenes were assigned to 286 pathways (Supplementary Table S1). Metabolic pathways had the largest number of unigenes (1459, 21.02%, ko01100), followed by biosynthesis of secondary metabolites (732, 10.55%, ko01110) and microbial metabolism in diverse environments (350, 5.04%, ko01120). Various pathways belonging to carbohydrate metabolism, amino acid metabolism, lipid metabolism, xenobiotics biodegradation and metabolism, etc. were also assigned to numerous unigenes.

### 2.3. Putative Genes Involved in DHA Biosynthesis

**Figure 4:**
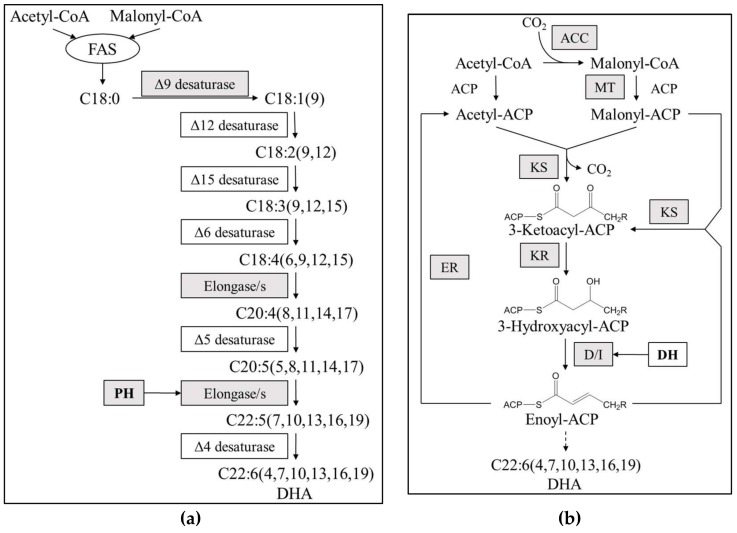
The fatty acid synthesis (FAS, **a**) and polyketide synthase (PKS, **b**) pathways for DHA biosynthesis. Shaded boxes show the putative genes detected from *Aurantiochytrium* sp. PKU#SW7 transcriptome; White boxes indicate genes not detected in the present study. Two candidate genes predicted to encode enzymes catalyzing fatty acid elongation (denoted as PH, **a**) in FAS pathway or functioning as dehydrase/isomerase (denoted as DH, **b**) in PKS pathway, were selected for functional analysis. ACC: Acetyl-CoA carboxylase; ACP: Acyl carrier protein; MT: Malonyl transferase; KS: Ketoacyl synthase; KR: Keto reductase; D/I: Dehydrase/isomerase; ER: Enoyl reductase.

Growing interest in thraustochytrids as potential sources for the production of high-value DHA, makes it meaningful to ascertain the DHA biosynthesis pathway in thraustochytrids strains [33,34]. Analysis of *Aurantiochytrium* sp. PKU#SW7 transcriptome under optimal DHA biosynthesis conditions [16] revealed several fatty acid desaturases and elongases encoding genes involved in the FAS pathway; however the critical Δ12, Δ6 and Δ4 desaturase genes essential for DHA synthesis were not observed (Figure 4a), implying incomplete FAS pathway in *Aurantiochytrium*. These findings were consistent with previous researches that the expected elongases and/or desaturases involved in FAS pathway failed to be detected through expressed sequence tags sequencing [21] or PCR based detection [19] from *Aurantiochytrium* strains, indicating possibility that the alternate PKS enzymes could be responsible for DHA synthesis in *Aurantiochytrium*. Indeed, 48 non-redundant sequences, which were related to the PKS pathway, were excavated in the present study (Table 1). These unigenes were homologs of ketoacyl synthase (KS), ketoacyl reductase (KR), malonyl CoA-acyl carrier protein transacylase (MAT), enoyl reductase (ER), dehydrase/isomerase (DH), etc., which are key components of polyketide synthesis (Figure 4b) [21]. These results further suggested that DHA synthesis in *Aurantiochytrium* is likely to occur through PKS pathway.

### 2.4. The Relative Expression Level of Putative Key Genes Involved in FAS or PKS Pathways under Various Cultivation Conditions

**Figure 5:**
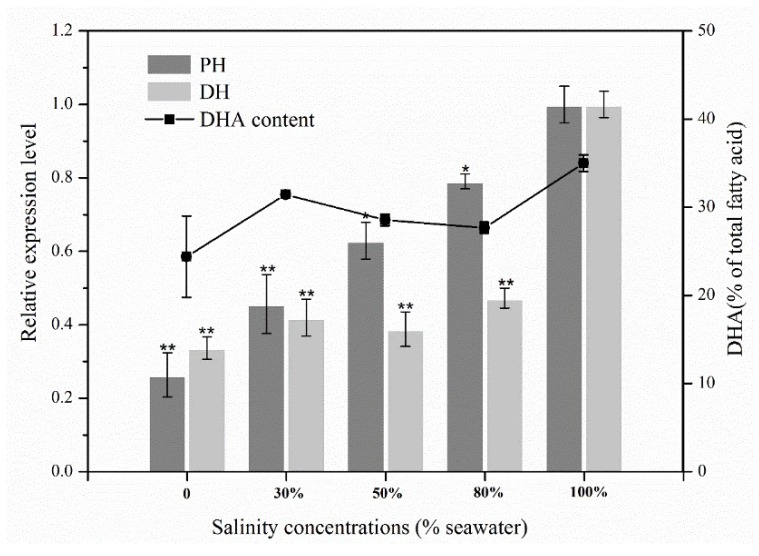
Relative expression level of PH and DH genes, and the DHA content (% of total fatty acid, represented by line [16]) of *Aurantiochytrium* sp. PKU#SW7 under different salinity conditions. Dark grey bar, PH gene; Light grey bar, DH gene; Line, DHA content. Independent-Samples *t*-test, ** (*p* < 0.001), * (*p* < 0.05).

To elucidate which pathway is responsible for DHA biosynthesis in *Aurantiochytrium* sp., two candidate genes predicted to encode the very-long-chain (3R)-3-hydroxyacyl-CoA dehydratase catalyzing fatty acid elongation from C20:5 to C22:5 (denoted as PH, Figure 4a) in FAS pathway or dehydrase/isomerase to warrant the precise location and stereospecific configuration of double bonds in the PUFAs (denoted as DH, Figure 4b) in PKS pathway were selected for further analysis. Both enzymes showed high similarity to their homologs in *Aurantiochytrium* sp. FCC1311 (Figure S1). The qRT-PCR (quantitative real-time PCR) performed under different salinity conditions demonstrated that significantly lower expression levels of both genes were observed when salinity decreased to less than 100% seawater (33.70 psu, Figure 5). Besides, PH and DH genes exhibited slightly different expression patterns (Figure 5). The expression level of PH gene increased gradually with the increase of salinity. The expression level of DH gene increased with salinity increasing from 0 to 30% seawater, then was constant in the salinity range from 30 to 80% seawater, and increased sharply when the salinity increased to 100% seawater (Figure 5). Moreover, the changing tendency of DH gene expression level showed good correlation with the *Aurantiochytrium* sp. DHA content across different salinity concentrations in accordance to our previous research [16], suggesting that the DH involved PKS pathway might contribute substantially for DHA synthesis in *Aurantiochytrium* sp. Additionally, the down-regulation of PH expression (Figure 5) was also combined with the lower DHA content [16] when salinity dropped below 100% seawater, indicating that the FAS pathway could also relate to DHA accumulation in *Aurantiochytrium* sp. to a certain extent.

### 2.5. Characterization of ΔPH or ΔDH Aurantiochytrium sp. Mutants

**Figure 6:**
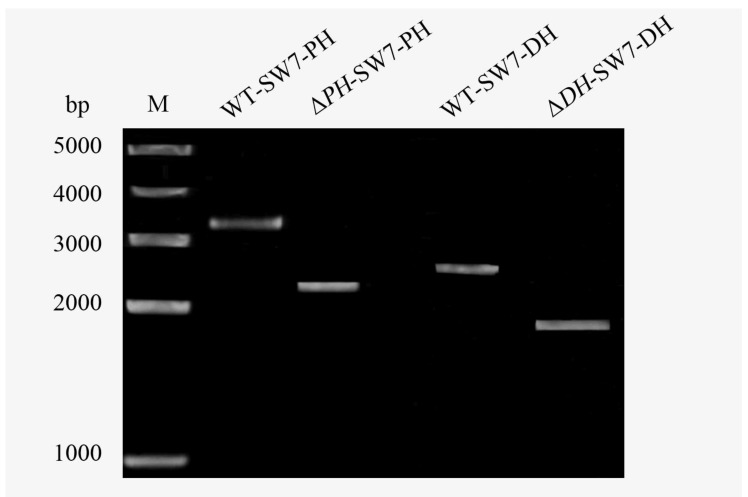
Colony PCR confirmation of the mutant strains (Δ*PH*-SW7 and Δ*DH*-SW7) and wild type strain (WT-SW7). Primer sets used for colony PCRs can be found in Table 2. The sizes of PCR fragments with primer set PHck-for/PHck-rev (for Δ*PH*-SW7 confirmation) are 3286 bp in wild type strain, and 2237 bp in Δ*PH*-SW7. The sizes of PCR fragments with primer set DHck-for/DHck-rev (for Δ*DH*-SW7 confirmation) are 2484 bp in wild type strain, and 1902 bp in Δ*DH*-SW7. M: molecular weight marker.

**Figure 7:**
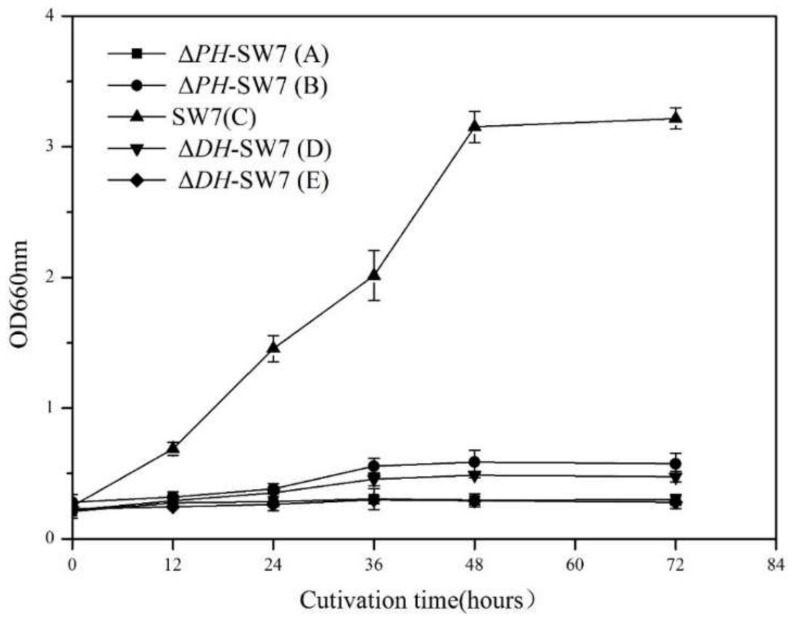
Time course growth profile of mutant strains (Δ*PH*-SW7 and Δ*DH*-SW7) as compared to wild type strain (SW7). A: M4 medium + 5 mg/mL hygromycin B; B: M4 medium + 5 mg/mL hygromycin B + 1 mg/mL DHA; C: M4 medium; D: M4 medium + 5 mg/mL G418 + 1 mg/mL DHA; E: M4 medium + 5 mg/mL G418.

To verify relative contribution of FAS and PKS pathway for DHA biosynthesis, the recombination cassettes (pYES2.1-PH-HygR, pYES2.1-DH-NeoR) were transformed independently into *Aurantiochytrium* sp. PKU#SW7 by electroporation. Two mutants, i.e., Δ*PH*-SW7 (lacking elongase activity for the elongation from C20:5 to C22:5, Figure 4) and Δ*DH*-SW7 (lacking dehydrase/isomerase activity, Figure 4 and Figure 6) were obtained. The initial research design for this study was to test the lipid and DHA accumulation of each mutant relative to the parental wild type, therefore revealing the impact of PH and DH genes on DHA synthesis. However, the cell densities of mutant strains were unexpectedly low that barely any biomass could be collected for further analysis (Figure 7). As shown in Figure 7, the cell growth of Δ*PH*-SW7 and Δ*DH*-SW7 were substantially lower than that of wild type when cultivated in M4 medium, indicating that the knock-out of PH or DH gene may cause severe negative effects on the growth and metabolism of *Aurantiochytrium sp.* As reported previously, auxotrophic mutants were obtained when a PUFA synthase was disrupted in *Schizochytrium*, showing that certain PUFAs were required for the complementation of auxotrophic mutants [18]. According to the KEGG annotation of *Aurantiochytrium* sp. transcriptome in the present study, PH and DH genes were predicted to be involved in FAS and PKS pathways, respectively, for PUFAs accumulation. Therefore, with the disruption of PH or DH gene, certain parts of PUFAs biosynthesis may be interrupted, resulting in auxotrophic *Aurantiochytrium* mutants. Besides, it is widely known that very long chain fatty acids (VLCFAs) with more than 18 carbons in length are the membrane components in eukaryotic cells. The lack of VLCFAs may cause reduction in the cell division, which consequently suppress the reproduction of *Aurantiochytrium* sp. On the other hand, it was reported that DHA plays a key role in cell membrane performance and serves as a readily available energy storage [35]. The accumulation of DHA in thraustochytrids was also believed to contribute significantly to the extensive production of plasma membrane system of the ectoplasmic net, which is crucial for the nutrient uptake of thraustochytrids and subsequently cell proliferation [35,36]. In the present study, the disruption of PH or DH gene may have resulted in hindered biosynthesis of DHA in *Aurantiochytrium* sp.; consequently little or even no DHA would be produced in the cells, which in turn dramatically limited the cell growth of *Aurantiochytrium* sp. (Figure 7). This corresponds to the results reported by Lippmeier et al. [18] that the PUFA synthase disrupted *Schizochytrium* auxotrophs grew significantly better when fed long chain PUFAs (C > 20) than those fed C18 PUFAs. Therefore, the overexpression of PH and/or DH genes in *Aurantiochytrium* sp. might be a potentially useful strategy to improve DHA content and relevant future studies will be of great interest and meaningful for DHA production through metabolic engineering. Furthermore, DHA was supplemented into the M4 medium, expecting the mutants to utilize the exogenous DHA source. After 48 hours of cultivation, the maximum OD660 values of Δ*PH*-SW7 and Δ*DH*-SW7 in medium supplemented with DHA were roughly twice higher than those in medium without DHA, reaching 0.588 and 0.495, respectively. Nevertheless, the biomass accumulation of these two mutants were much lower than that of wild type and insufficient for any further analysis (Figure 7). These results suggest that exogeneous DHA can only partially rescue either of the knockout strains, probably because intracellular DHA rather than exogenous DHA is required for thraustochytrid cell growth or because the strain *Aurantiochytrium* sp. is unable to effectively uptake free fatty acids since the wild type *Aurantiochytrium* sp. was unable to uptake the supplemented 13C labelled palmitate in the medium (data not shown). Moreover, it can be speculated that both PH and DH genes are engaged in PUFAs/DHA biosynthesis, and are essential for cell growth of *Aurantiochytrium* sp. Future work to explore the corresponding roles and contributions of FAS and PKS pathways in DHA biosynthesis would be an interesting subject. 

## 3. Materials and Methods 

### 3.1. Microbial Material

*Aurantiochytrium* sp. PKU#SW7, originally isolated from the coastal water of Southern China (22°31′32.632″ N, 114°28′40.185″ E) [24], was used in the present study. The isolate culture was maintained on modified Vishniac’s (MV) agar plate [37] at 28 °C and sub cultured every 15–30 days. To collect biomass for RNA isolation, the strain was cultivated in a fermenter under the optimized conditions, as reported in our previous research [16]. The collected biomass was snap-frozen with liquid nitrogen and maintained at −80 °C until RNA extraction.

### 3.2. RNA Extraction, cDNA Construction, and RNA-seq

The total RNA of *Aurantiochytrium* sp. PKU#SW7 was extracted with RNAEx^TM^ total RNA isolation solution (Generay, Shanghai, China) according to the manufacturer’s guidelines. Briefly, samples were homogenized in 1 mL RNAEx^TM^ using a MP homogenizer (MP FastPrep^®^-24, Santa Ana, CA, USA). Then, 200 μL of chloroform was added and the mixtures were incubated for 2 min at room temperature. After centrifugation at 12,000 g for 10 min, the aqueous phase was transferred to a fresh tube, and 500 μL of isopropyl alcohol was added for RNA precipitation and recovery through centrifugation at 12,000 g for 10 min. Afterwards, 1 mL of 75% ethanol was added for RNA washing and the RNA pellet was dissolved in DEPC (diethyl pyrocarbonate) treated water. The integrity of RNA was detected by agarose gel electrophoresis and the concentration was estimated by a UV-Vis spectrophotometer Nanophotometer P300 (Implen, Munich, Germany) and Agilent 2100 bioanalyzer (Agilent, Santa Clara, CA, USA). RNA samples with OD260/280 ≥ 1.8, OD260/230 ≥ 1.8, and RIN > 9.5 were selected for cDNA library construction. Finally, the cDNA libraries were sequenced at Beijing Genomic Institute (BGI)-Shenzhen, China, using Illumina Hiseq^TM^ 2000 according to the manufacturer’s instructions (Illumina, San Diego, CA, USA).

### 3.3. Transcriptome Assembly and Analysis

Sequences containing adaptor, with more than 5% of unknown bases, or with greater than 20% of low quality bases (base with quality value ≤ 10) were filtered out first. De novo assembly of the clean reads was then performed by the short reads assembling program Trinity. Clean reads with a certain overlap were first combined by paired-end mapping to form longer fragments, i.e., contigs, which were further connected into unigenes that could not be extended on either end. The unigenes obtained by Trinity were further processed for sequence splicing and redundancy removal, and were finally split into two classes: clusters (similarity > 70%) and singletons. The sequence orientations and coding sequences (CDS) predictions were performed by blastx against different databases (*E* value < 1×10^−5^), within which a following priority was taken: the NCBI nonredundant protein (Nr) database, the Swiss-Prot protein database, the Kyoto Encyclopedia of Genes and Genomes (KEGG) pathway database [38] and the Cluster of Orthologous Groups (COG) database. When an unigene did not align to any of the databases, ESTScan was used to predict the sequence direction [39]. 

Functional annotation of all unigenes was performed by blastx against the above protein databases, and blastn against Nt database (*E* value < 1×10^−5^). Based on the Nr annotation, Blast2GO program [40] was applied for gene ontology (GO) annotation of all unigenes, and the WEGO software (version 1.0, Beijing, China) was further used to cluster the unigenes according to their function [41].

### 3.4. Candidate Genes

According to the CDS annotation, two candidate unigenes predicted to encode very-long-chain (3R)-3-hydroxyacyl-CoA dehydratase (denoted as PH, 2742 bp, accession number: MH469721) and dehydrase/isomerase (denoted as DH, 859 bp, accession number: MH469720), which are potentially involved in the two distinct PUFA biosynthesis pathways (FAS and PKS pathways, respectively) were selected for further functional analysis. Tail PCR with a GenomeWalker Kit™ (Takara Bio, Kusatsu, Shiga Prefecture, Japan) was applied to attain the flanking sequences of these two genes for homologous recombination.

### 3.5. Quantitative Real-Time PCR (qRT-PCR)

The relative expression level of PH and DH genes under various cultivation conditions were analyzed by quantitative real-time PCR (qRT-PCR). According to our previous results [16], *Aurantiochytrium* sp. PKU#SW7 was cultured at 28 °C with different salinity values of 0%, 30%, 50%, 80% or 100% seawater (0, 10.11, 16.85, 26.96 or 33.70 psu). The total RNA was extracted from 15 mL of 2 day-old cultures using a Trizol reagent (Sigma Aldrich, St. Louis, MO, USA). RNase-free DNase was used to remove genomic DNA from the RNA samples. The reverse transcription reaction was carried out using PrimerScript^TM^ RT reagent Kit with gDNA Eraser (TaKaRa Bio, Kusatsu, Shiga Prefecture, Japan). The resultant cDNA molecules were then used for the amplification of β-actin gene, which was used as an internal control by gene-specific primers: BF and BR (Table 2); primer sets PHF/PHR and DHF/DHR (Table 2) were applied for the amplification of PH and DH genes, separately. The qRT-PCR was conducted with SYBR^®^Premix Ex Taq^TM^ (TaKaRa Bio, Kusatsu, Shiga Prefecture, Japan) in CFX96 Touch Real Time PCR system (Bio-Rad, Hercules, CA, USA). Three independent biological replicates of each sample were performed, and each PCR reaction included three technical replicates. Changes in gene expression were calculated using the 2^−ΔΔCt^ method. The data were tested by Independent-Samples t-test with SPSS version 19.0 software (SPSS Inc., Chicago, IL, USA).

### 3.6. Antibiotics Screening

Pre-cultured 2 day-old *Aurantiochytrium* sp. PKU#SW7 was inoculated in 5 mL of M4 medium containing various concentrations of antibiotics: G418 (1, 2, 3 mg/mL), hygromycin B (1, 2, 3 mg/mL) and blasticidin (50, 100, 200 µg/mL). After 4 days of shaking cultivation at 25 °C and 150 rpm, the inhibition effect was determined by measuring cellular growth with a UV-Vis spectrophotometer P300 (Implen, Munich, Germany) at 660 nm (OD660). Afterwards, the minimal inhibitory concentrations (MICs) of each antibiotic were further determined by streaking *Aurantiochytrium* sp. PKU#SW7 culture on MV agar plate containing different antibiotics at 25 °C. Neomycin and hygromycin phosphotransferase confer resistance to the neomycin analogue G418 and hygromycin B, respectively.

### 3.7. Construction of HygR and NeoR Recombination Cassettes

**Figure 8:**
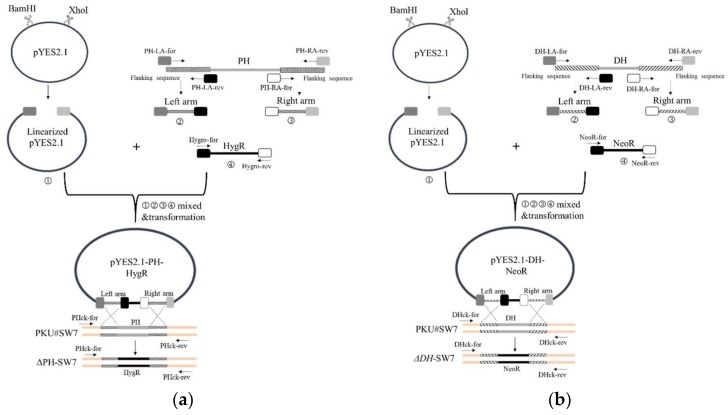
Schematic diagram showing the strategy for constructing gene knock-outs *Aurantiochytrium* (**a**: Δ*PH* -SW7; **b**: Δ*DH*-SW7) by homologous recombination.

The resistance genes (HygR and NeoR) were synthesized with optimized codon usage into pUC57-Amp-HygR and pUC57-Amp-NeoR. Primer sets Hygro-for/Hygro-rev and NeoR-for/NeoR-rev were used for HygR and NeoR amplification and cloning, respectively (Table 2). *S. cerevisiae*–*E. coli* shuttle vector pYES2.1 (Life Technologies, Carlsbad, CA, USA) was digested with XhoI and BamHI and used as the framework for formation of recombination cassettes. The upstream (LA) and downstream (RA) flanking sequences over 550 bp from 5’ end and 3’ end of target genes respectively were obtained for replacement of PH and DH genes in the recombination cassettes. In detail, specific primer sets (Table 2) were designed to generate products comprising 22–25 bp overhangs to the end of the linearized vector at the beginning, followed by the upstream flanking sequences of target genes; and 22–25 bp overhangs to the beginning of the resistance gene (LA), or comprising 22–25 bp homologous sequences to the end of the resistance genes at the beginning, followed by the downstream flanking sequences of target genes, and 22–25 bp overhangs to the linearized vector (RA) (Figure 8). All PCR reactions were carried out by Phusion *Pfu* DNA polymerase (NEB, Ipswich, MA, USA).

Subsequently, the equimolar quantities of amplified fragments and predigested vector were mixed together and transformed into *S. cerevisiae* using LiAc method essentially as described by Gietz et al. [42] for in vivo construct assembly. Transformation mixture was plated on SC-U (SC minimal media lacking uracil) (Takara Bio, Kusatsu, Shiga Prefecture, Japan). The successfully built plasmids were isolated from yeast using Zymoprep Yeast Plasmid Miniprep II (Zymo Research, Irvine, CA, USA), amplified in *E. coli* DH5ɑ, and verified by sequencing by BGI Shenzhen (China).

### 3.8. Transformation of Aurantiochytrium sp. PKU#SW7

To generate PH or DH deletion mutants, the constructed plasmids (pYES2.1-PH-HygR, pYES2.1-DH-NeoR, Figure 8) with antibiotic resistance gene were introduced into *Aurantiochytrium* sp. PKU#SW7 by electroporation. An aliquot of 2-day old *Aurantiochytrium* sp. cultures were harvested and the cells were subsequently resuspended in 1 M sorbitol solution [43]. For electroporation, up to 6 µg of plasmids DNA were mixed with 40 µL of thraustochytrids cells. The mixture solution was transferred to a 0.2-cm-gap cuvette with 80 µL of electroporation buffer (Bio-Rad, Hercules, CA, USA) and then electroporated by a Gene Pulser apparatus (Bio-Rad, Hercules, CA, USA). The pulse conditions used were as following: 50 µF, 50 Ω, 3.0 kV/cm 2 times. Immediately after the pulse, 1 mL of M4 medium was added to the cuvette, and the mixture was transferred to a sterile 1.5 mL tube. After incubation at 28 °C overnight, the mixture culture was spread on a MV plate with corresponding antibiotic and DHA (1 mg/mL), and then continued to incubate at 28 °C until colonies appeared. Colony PCR was used for identifying successful knock-out strains (Figure 6); single colonies were screened for the recombination cassette using primer set PHck-for/PHck-rev and DHck-for/DHck-rev (Table 2, Figure 8), cloned and confirmed by sequencing. Positive colonies were subsequently cultured in M4 medium with corresponding antibiotic and/or DHA (1 mg/mL) in 96-well plates for further characterization. 

## 4. Conclusions

The transcriptome of thraustochytrids strain *Aurantiochytrium* sp. PKU#SW7 was analyzed in the present study. Both FAS and PKS pathway related genes for PUFAs biosynthesis were identified. Of the two pathways, the FAS was incomplete and lacking key desaturases and elongases. Quantitative real-time PCR of two putative genes (i.e., very-long-chain (3R)-3-hydroxyacyl-CoA dehydratase and dehydrase/isomerase encoding genes) involved in FAS and PKS pathways, respectively, revealed that the varying tendency of their relative expression level was roughly consistent with DHA content variation under different salinity conditions. Furthermore, disruption of either of these two genes in *Aurantiochytrium* sp. resulted in poor cell growth, probably due to the interruption of certain parts of the PUFAs synthesis, or little or no intracellular DHA accumulation, suggesting that both PH and DH genes are engaged in PUFAs/DHA biosynthesis and are essential for cell growth of *Aurantiochytrium* sp.

## Figures and Tables

**Table 1:** Putative unigenes identified from the *Aurantiochytrium* transcriptome that are homologous to polyketide synthases (PKS).

Putative Enzymes	Number of Unigenes
Ketoacyl synthase	8
3-oxoacyl-[acyl carrier protein] synthase	3
Keto reductase	15
Malonyl CoA-acyl carrier protein transacylase	1
Malonyl transferase	3
Dehydrase/isomerase	2
Enoyl reductase	4
Hydroxyacyl dehydrogenase	6
3-hydroxydecanoyl-[acyl carrier protein] dehydratases	4
Phosphopantetheinyl transferase	2

**Table 2:** All primers used in this research.

Primer Name	Sequence 5′→3′	Amplicon Length
PH-LA-for	CTATAGGGAATATTAAGCTCGCCCTTCTTAGTCTCCGCTTGCGTCGTC	575 bp
PH-LA-rev	GTAGCAGTGAGTTCGGGCTTCTTCATCGCCAGGTTGTAGGCAATGAGG	
PH-RA-for	CTACTCGCCCTCGCGCTAAGGAGTAAGGTTTGTCATTACGCCAGTTGT	584 bp
PH-RA-rev	AATGGGTGACCTCGAAGCTCGCCCTCTACGCCACCAGTCCTAACAAG	
DH-LA-for	CTATAGGGAATATTAAGCTCGCCCTTTTCTAAGGCCGGCTATGTATGC	559 bp
DH-LA-rev	ACCGTCCTGCTCAATAGCAGACATGTCCAGGAAGCATCCGACACAC	
DH-RA-for	CCGTCTCCTCGATGAGTTTTTTTAACTTTAAGAAAGGAAGCAATGAGCC	545 bp
DH-RA-rev	AATGGGTGACCTCGAAGCTCGCCCTCTAAATGATACAGCCTTTGTTCGT	
PHck-for	CTTCTTAGTCTCCGCTTGCGTCGTC	Δ*PH*-SW7: 2237 bp SW7: 3286 bp
PHck-rev	CTCTACGCCACCAGTCCTAACAAG	
DHck-for	TTCTAAGGCCGGCTATGTATGC	Δ*DH*-SW7: 1902 bp SW7: 2484 bp
DHck-rev	TCTAAATGATACAGCCTTTGTTCG	
Hygro-for	ATGAAGAAGCCCGAACTCACTGC	1078 bp
Hygro-rev	TTACTCCTTAGCGCGAGGGCGAGTA	
NeoR-for	ATCTCATGACCAAAATCCCTTAACGTG	798 bp
NeoR-rev	TTAAAAAAACTCATCGAGGAGACGGT	
BF	ATGGCTGACGACGAAGTTCAAGC	175 bp
BR	CCTCATCACCGACATAGGC	
PHF	TGGTGCTAGGAGCAACGTTGCTAG	139 bp
PHR	TTCTGGCCTGAAGCTCAACAACTC	
DHF	GGCAATCATAATAAGCTTCCTTTCACCTTGC	133 bp
DHR	CATCTTTAAGAAAGGAAGCAATGAGCCG	

## Supplementary Materials

The following are available online at http://www.mdpi.com/1660-3397/16/9/310/s1. Figure S1: Sequence alignment of very-long-chain (3R)-3-hydroxyacyl-CoA dehydratase (PH, **a**) and dehydrase/isomerase (DH, **b**) enzyme by ClustalW. (**a**) Identification of very-long-chain (3R)-3-hydroxyacyl-CoA dehydratase. FCC1311: *Aurantiochytrium* sp. FCC1311 very-long-chain (3R)-3-hydroxyacyl-CoA dehydratase (GBG31679.1); (**b**) Identification of dehydrase/isomerase. FCC1311: *Aurantiochytrium* sp. FCC1311 dehydrase/isomerase (GBG29999.1). Table S1: KEGG pathways assignment. A total of 6941 unigenes were assigned to 286 pathways.

## References

[1] Sparrow F.K. (1936). Biological observations on the marine fungi of woods hole waters. Biol. Bull..

[2] Raghukumar S., Balasubramanian R. (1991). Occurrence of thraustochytrids fungi in coral and coral mucus. Indian J. Mar. Sci..

[3] Bongiorni L., Raghukumar C. (2012). Thraustochytrids, a neglected component of organic matter decomposition and food webs in marine sediments. Biology of Marine Fungi.

[4] Raghukumar S. (1992). Bacterivory: A novel dual role for thraustochytrids in the sea. Mar. Biol..

[5] Bongiorni L., Pusceddu A., Danovaro R. (2005). Enzymatic activities of epiphytic and benthic thraustochytrids involved in organic matter degradation. Aquat. Microb. Ecol..

[6] Miller M.R., Nichols P.D., Carter C.G. (2007). Replacement of fish oil with thraustochytrid *Schizochytrium* sp. L. oil in Atlantic salmon parr (*Salmo salar* L.) diets. Comp. Biochem. Phys. A.

[7] Shah M.R., Lutzu G.A., Alam A., Sarker P., Kabir Chowdhury M.A., Parsaeimehr A., Liang Y., Daroch M. (2018). Microalgae in aquafeeds for a sustainable aquaculture industry. J. Appl. Phycol..

[8] Lewis T.E., Nichols P.D., McMeekin T.A. (1999). The biotechnological potential of thraustochytrids. Mar. Biotechnol..

[9] Raghukumar S. (2008). Thraustochytrid Marine Protists: Production of PUFAs and Other Emerging Technologies. Mar. Biotechnol..

[10] Gupta A., Wilkens S., Adcock J.L., Puri M., Barrow C.J. (2013). Pollen baiting facilitates the isolation of marine thraustochytrids with potential in omega-3 and biodiesel production. J. Ind. Microbiol. Biot..

[11] Chang K.J.L., Dunstan G.A., Abell G.C.J., Clementson L.A., Blackburn S.I., Nichols P.D., Koutoulis A. (2012). Biodiscovery of new Australian thraustochytrids for production of biodiesel and long-chain omega-3 oils. Appl. Microbiol. Biotechnol..

[12] Marchan L.F., Chang K.J.L., Nichols P.D., Mitchell W.J., Polglase J.L., Gutierrez T. (2018). Taxonomy, ecology and biotechnological applications of thraustochytrids: A review. Biotechnol. Adv..

[13] Yaguchi T., Tanaka S., Yokochi T., Nakahara T., Higashihara T. (1997). Production of high yields of docosahexaenoic acid by *Schizochytrium* sp. strain SR21. J. Am. Oil Chem. Soc..

[14] Chi Z., Liu Y., Frear C., Chen S. (2009). Study of a two-stage growth of DHA-producing marine algae *Schizochytrium limacinum* SR21 with shifting dissolved oxygen level. Appl. Microbiol. Biotechnol..

[15] Chaung K.-C., Chu C.-Y., Su Y.-M., Chen Y.-M. (2012). Effect of culture conditions on growth, lipid content, and fatty acid composition of *Aurantiochytrium mangrovei* strain BL10. AMB Express.

[16] Liu Y., Tang J., Li J., Daroch M., Cheng J.J. (2014). Efficient production of triacylglycerols rich in docosahexaenoic acid (DHA) by osmo-heterotrophic marine protists. Appl. Microbiol. Biotechnol..

[17] Matsuda T., Sakaguchi K., Hamaguchi R., Kobayashi T., Abe E., Hama Y., Hayashi M., Honda D., Okita Y., Sugimoto S. (2012). Analysis of Δ12-fatty acid desaturase function revealed that two distinct pathways are active for the synthesis of PUFAs in *T. aureum* ATCC 34304. J. Lipid Res..

[18] Lippmeier J.C., Crawford K.S., Owen C.B., Rivas A.A., Metz J.G., Apt K.E. (2009). Characterization of Both Polyunsaturated Fatty Acid Biosynthetic Pathways in *Schizochytrium* sp.. Lipids.

[19] Nagano N., Sakaguchi K., Taoka Y., Okita Y., Honda D., Ito M., Hayashi M. (2011). Detection of Genes Involved in Fatty Acid Elongation and Desaturation in Thraustochytrid Marine Eukaryotes. J. Oleo Sci..

[20] Qiu Xiao H.H., MacKenzie Samuel L. (2001). Identification of a Δ4 fatty acid desaturase from *thraustochytrium* sp. involved in the biosynthesis of docosahexanoic acid by heterologous expression in *saccharomyces cerevisiae* and *brassica juncea*. J. Biol. Chem..

[21] Metz J.G., Roessler P., Facciotti D., Levering C., Dittrich F., Lassner M., Valentine R., Lardizabal K., Domergue F., Yamada A. (2001). Production of polyunsaturated fatty acids by polyketide synthases in both prokaryotes and eukaryotes. Science.

[22] Hertwech C. (2009). The biosynthetic logic of polyketide diversity. Angew. Chem. Int. Ed..

[23] Ratledge C. (2004). Fatty acid biosynthesis in microorganisms being used for Single Cell Oil production. Biochimie.

[24] Liu Y., Singh P., Sun Y., Luan S., Wang G. (2014). Culturable diversity and biochemical features of thraustochytrids from coastal waters of Southern China. Appl. Microbiol. Biotechnol..

[25] Ma Z., Tan Y., Cui G., Feng Y., Cui Q., Song X. (2015). Transcriptome and gene expression analysis of DHA producer *Aurantiochytrium* under low temperature conditions. Sci. Rep..

[26] Wang Z., Gerstein M., Snyder M. (2009). RNA-Seq: A revolutionary tool for transcriptomics. Nat. Rev. Genet..

[27] Chen W., Zhou P.-P., Zhang M., Zhu Y.-M., Wang X.-P., Luo X.-A., Bao Z.-D., Yu L.-J. (2016). Transcriptome analysis reveals that up-regulation of the fatty acid synthase gene promotes the accumulation of docosahexaenoic acid in *Schizochytrium* sp. S056 when glycerol is used. Algal Res..

[28] Li J., Han D., Wang D., Ning K., Jia J., Wei L., Jing X., Huang S., Chen J., Li Y., Hu Q., Xu J. (2014). Choreography of transcriptomes and lipidomes of *Nannochloropsis* reveals the mechanisms of oil synthesis in microalgae. Plant Cell.

[29] Li D., Deng Z., Qin B., Liu X., Men Z. (2012). De novo assembly and characterization of bark transcriptome using Illumina sequencing and development of EST-SSR markers in rubber tree (*Hevea brasiliensis* Muell. Arg.). BMC Genom..

[30] Wei W., Qi X., Wang L., Zhang Y., Hua W., Li D., Lv H., Zhang X. (2011). Characterization of the sesame (*Sesamum indicum* L.) global transcriptome using Illumina paired-end sequencing and development of EST-SSR markers. BMC Genom..

[31] Parchman T.L., Geist K.S., Grahnen J.A., Benkman C.W., Buerkle C.A. (2010). Transcriptome sequencing in an ecologically important tree species: Assembly, annotation, and marker discovery. BMC Genom..

[32] Huang J., Jiang X., Zhang X., Chen W., Tian B., Shu Z., Hu S. (2008). Expressed sequence tag analysis of marine fungus *Schizochytrium* producing docosahexaenoic acid. J. Biotechnol..

[33] Gupta A., Barrow C.J., Puri M. (2012). Omega-3 biotechnology: Thraustochytrids as a novel source of omega-3 oils. Biotechnol. Adv..

[34] Ryu B.-G., Kim K., Kim J., Han J.-I., Yang J.-W. (2013). Use of organic waste from the brewery industry for high-density cultivation of the docosahexaenoic acid-rich microalga, *Aurantiochytrium* sp. KRS101. Bioresour. Technol..

[35] Jain R., Raghukumar S., Sambaiah K., Kumon Y., Nakahara T. (2007). Docosahexaenoic acid accumulation in thraustochytrids: Search for the rationale. Mar. Biol..

[36] Gladyshev M.I., Sushchik N.N., Makhutova O.N. (2013). Production of EPA and DHA in aquatic ecosystems and their transfer to the land. Prostag. Other Lipid Mediat..

[37] Damare V., Raghukumar S. (2006). Morphology and physiology of the marine straminipilan fungi, the aplanochytrids isolated from the equatorial Indian Ocean. Indian J. Mar. Sci..

[38] Kanehisa M., Araki M., Goto S., Hattori M., Hirakawa M., Itoh M., Katayama T., Kawashima S., Okuda S., Tokimatsu T., Yamanishi Y. (2008). KEGG for linking genomes to life and the environment. Nucleic Acids Res..

[39] Iseli C., Jongeneel C.V., Bucher P. (1999). ESTScan: A program for detecting, evaluating, and reconstructing potential coding regions in EST sequences. Proc. Int. Conf. Intell. Syst. Mol. Biol..

[40] Conesa A., Gotz S., Garcia-Gomez J.M., Terol J., Talon M., Robles M. (2005). Blast2GO: A universal tool for annotation, visualization and analysis in functional genomics research. Bioinformatics.

[41] Ye J., Fang L., Zheng H., Zhang Y., Chen J., Zhang Z., Wang J., Li S., Li R., Bolund L. (2006). WEGO: A web tool for plotting GO annotations. Nucleic Acids Res..

[42] Gietz R.D., Schiestl R.H. (2007). High-efficiency yeast transformation using the LiAc/SS carrier DNA/PEG method. Nat. Protoc..

[43] Becker D.M., Guarente L. (1991). High-efficiency transformation of yeast by electroporation. Method Enzymol..

