# Supplementary material for: Transcriptomic Profiling and Gene Disruption Revealed that Two Genes Related to PUFAs/DHA Biosynthesis May be Essential for Cell Growth of Aurantiochytrium sp
Source: Mar Drugs. 2018 Sep 1;16(9):310. doi: 10.3390/md16090310 (PMC6164183; doi:10.3390/md16090310)
Supplement: Supplementary file 1 [file marinedrugs-16-00310-s001.pdf]

```
          1        10        20        30        40        50        60
SW7-PH    MSAAKMYLIAYNLASCAGWAYVWVEVAKAIMNGASAAQLWQESSNVLAVVQSLAALEVLH
FCC1311   .MATKMYLVAYNLASCAGWAYVWTQTVKTLLNGGKAGMLWNEASDVLAVVQSLAALEIVH

                   70        80        90       100       110       120
SW7-PH    SIIRLVKSPVMTVLMQVSSRLLVLWLYTWPASACHSHWSLLLMVGSWASVEVPRYLFYAL
FCC1311   SLLRLVKSPVFTVFMQVNSRLIVLWLYTWQAAACHSHWSLLLMVGSWATVEVPRYLFYAL

                  130       140       150       160       170       180
SW7-PH    NLLPAFQGSKMPYPLFWLRYSLFMVLYPTGITGELLQMYVALTTYYTFGSAWERLLFVIP
FCC1311   NLLPSFQGSKMPYPLFWLRYSLFMVLYPTGITGELVQMYVALSTHYTFNTAWERFLFVFP

                  190       200       210       220       230
SW7-PH    ILTYPPCGPFMVLNMWKNRKSQFRKRSQELAAAKD..GGAPQKKVDGLVWPVTNDATGER
FCC1311   LIAYPPASPFMVLNMWKNRKSQFRKRAQELAAAKEEGGASAKKAVSGLVWPVTNDATGER

         240       250       260       270       280       290
SW7-PH    STSVTNQSIWEYAVANVDPEAAAAVRKTRKWRFGYIKHIETQIRVSLRSKENALQVARDG
FCC1311   STSVTNQSIWEYAVSGADADAAAAVRKTRKWRFGYLRHIESQVRISLRSKETALQIARDG

         300       310       320       330       340       350
SW7-PH    LKRAHEVFEFVRDGKAMSLAQAMETYKGSYETGFIKGEGKRSVKEARVLYNGKTLVGDEL
FCC1311   LARAHEAFEFVRDGKATSLAEAMDKYKGSYETGFIKGEGKREVKEARVLYKGQTLVGDAL

         360       370       380       390       400       410
SW7-PH    VAQLDKWVTEGVIEPSAGDAVKKCIEHPEWYDLSDRYFVLLGATSAMGPLDLLLQCGANV
FCC1311   VAQLEKWVSEGVIEPSAGDAVKQCIAHPEWYDLSDRYFVLLGATSAMGPLDLLLQCGANV

         420       430       440       450       460       470
SW7-PH    VGIDLDRSPIWKKLIEKVRTSPGTLTFPLKKAQTLLKTDEELYENAGANLLGATPEIANW
FCC1311   IGIDLDRAPIWEKLINKVRASPGTLTFPLSKPQASLKTDADLFAHAGANLLGATPEIANW

         480       490       500       510       520       530
SW7-PH    LVGVCPGKDLTIGNYTYLDGALHVQLSIACDAIMQKVLAQRKTSTSLAFLLTPTDVYMIN
FCC1311   LVGVCPGQDLTIGNYTYLDGALHVQLSIACDAIMQKVLAKRSSSTSLAFLLTPTDVYMIN

         540       550       560       570       580       590
SW7-PH    EDAYEAAKENYKSAPAWQKLLEKIMGKNDMVSNVLKPAEGSGLKLNNSVVSHQGPNYSLA
FCC1311   EDAFEVAKANYKAAPAWQKALEKVMGKNDMVCNVLKPADGSGLKLSNAVVSAQGPNYSLA

         600       610       620       630       640       650
SW7-PH    KRIQQWRCLLAHEEGHTVASNVAPSTSTASVTSNPLFAAAYAGFELFKALEVFRPETSSS
FCC1311   KRIQQWRCIIAHSEGHTVSSNVAPSTSTASVTSNPLFAAAYAGFKLFKALEVFRPETSSS

         660       670       680       690       700       710
SW7-PH    LMLALLINDVRNPESVANPKSTVSSKMKNPLELFAHNAAHGGSFRSPYSIGSIGTVSVLY
FCC1311   LMLALLINDIRNPESISNPKSAVAAKMANPLELFAHNAAHGGSFRCPYSVGTIGTVSVLY

         720       730       740       750
SW7-PH    YFISNYWFVITPVVGAAAYTVNFVATGAHPGLNASK
FCC1311   YFIGNYWFAALPVVGLTAYTVSFVATGARPGLAAKQ
```

(a)

```
          1        10        20        30        40        50        60
SW7-DH    MDQIFIYIEALPVCRMLPGLGQALRGGRLLRRSVPGLELRSTSANRANTSQGSLNLHKDP
FCC1311   .....................................................MSGTSRG

                   70        80        90       100       110       120
SW7-DH    EKGISTAAQTIILAKRTMSTTKKERNQRENYRYWLPIQTRWGDNDMYGHVNNVVYYAYFD
FCC1311   TLTATAATKTTRGVRAASTSAKPERNQREDYRYWLPIQTRWGDNDMYGHVNNVVYYAYFD

                  130       140       150       160       170       180
SW7-DH    SIVNHFLISEAGLDPMTSDAIGLCIESYCNYYASLEYPEIIEAGLFVSHLGNSSLKYEVG
FCC1311   SVVNHFLIKEAGLKPMESDAIGLCIESYCNYYAPLEYPEVVEAGLFVSHVGRSSVKYEVG

                  190       200       210       220
SW7-DH    IFKKGSNEPAAHGHFVHVFVDRTTRRPVKIPEQIASKVKGSLL
FCC1311   IFKHNSNEQAAHGHFVHVFVDRDTRKPVPIPDAIMSKIKGSLL
```

(b)

Figure S1: Sequence alignment of very-long-chain (3R)-3-hydroxyacyl-CoA dehydratase (PH, a) and dehydrase/isomerase (DH, b) enzyme by ClustalW. (a) Identification of very-long-chain (3R)-3-hydroxyacyl-CoA dehydratase. FCC1311: *Aurantiochytrium* sp. FCC1311 very-long-chain (3R)-3-hydroxyacyl-CoA dehydratase (GBG31679.1); (b) Identification of dehydrase/isomerase. FCC1311: *Aurantiochytrium* sp. FCC1311 dehydrase/isomerase (GBG29999.1).

Table S1. KEGG pathways assignment. A total of 6941 unigenes were assigned to 286 pathways.

Level 1	Level 2	Level 3	Number of unigenes	Pathway ID
Metabolism	Global map	Metabolic pathways	1459 (21.02%)	ko01100
		Biosynthesis of secondary metabolites	732 (10.55%)	ko01110
		Microbial metabolism in diverse environments	350 (5.04%)	ko01120
	Carbohydrate metabolism	Amino sugar and nucleotide sugar metabolism	334 (4.81%)	ko00520
		Starch and sucrose metabolism	268 (3.86%)	ko00500
		Glycolysis / Gluconeogenesis	92 (1.33%)	ko00010
		Pyruvate metabolism	84 (1.21%)	ko00620
		Butanoate metabolism	81 (1.17%)	ko00650
		Inositol phosphate metabolism	69 (0.99%)	ko00562
		Fructose and mannose metabolism	66 (0.95%)	ko00051
		Propanoate metabolism	62 (0.89%)	ko00640
		Pentose and glucuronate interconversions	42 (0.61%)	ko00040
		Citrate cycle (TCA cycle)	42 (0.61%)	ko00020
		Glyoxylate and dicarboxylate metabolism	32 (0.46%)	ko00630
		Pentose phosphate pathway	25 (0.36%)	ko00030
		Ascorbate and aldarate metabolism	22 (0.32%)	ko00053
		Galactose metabolism	14 (0.2%)	ko00052
		C5-Branched dibasic acid metabolism	5 (0.07%)	ko00660
	Nucleotide metabolism	Purine metabolism	206 (2.97%)	ko00230
	Amino acid metabolism	Lysine degradation	86 (1.24%)	ko00310
		Valine, leucine and isoleucine degradation	80 (1.15%)	ko00280

	Tryptophan metabolism	77 (1.11%)	ko00380
	Arginine and proline metabolism	73 (1.05%)	ko00330
	Glycine, serine and threonine metabolism	70 (1.01%)	ko00260
	Alanine, aspartate and glutamate metabolism	62 (0.89%)	ko00250
	Tyrosine metabolism	61 (0.88%)	ko00350
	Cysteine and methionine metabolism	51 (0.73%)	ko00270
	Histidine metabolism	38 (0.55%)	ko00340
	Phenylalanine metabolism	32 (0.46%)	ko00360
	Phenylalanine, tyrosine and tryptophan biosynthesis	25 (0.36%)	ko00400
	Lysine biosynthesis	22 (0.32%)	ko00300
	Valine, leucine and isoleucine biosynthesis	18 (0.26%)	ko00290
Lipid metabolism	Fatty acid metabolism	83 (1.2%)	ko00071
	Glycerophospholipid metabolism	69 (0.99%)	ko00564
	Glycerolipid metabolism	48 (0.69%)	ko00561
	Sphingolipid metabolism	44 (0.63%)	ko00600
	Biosynthesis of unsaturated fatty acids	32 (0.46%)	ko01040
	Fatty acid biosynthesis	30 (0.43%)	ko00061
	Linoleic acid metabolism	30 (0.43%)	ko00591
	Steroid biosynthesis	29 (0.42%)	ko00100
	Fatty acid elongation	26 (0.37%)	ko00062
	Steroid hormone biosynthesis	18 (0.26%)	ko00140
	Ether lipid metabolism	17 (0.24%)	ko00565
	Synthesis and degradation of ketone bodies	16 (0.23%)	ko00072
	Primary bile acid biosynthesis	15 (0.22%)	ko00120
	Arachidonic acid metabolism	13 (0.19%)	ko00590

	alpha-Linolenic acid metabolism	9 (0.13%)	ko00592
Energy metabolism	Oxidative phosphorylation	81 (1.17%)	ko00190
	Methane metabolism	53 (0.76%)	ko00680
	Nitrogen metabolism	42 (0.61%)	ko00910
	Carbon fixation in photosynthetic organisms	27 (0.39%)	ko00710
	Carbon fixation pathways in prokaryotes	27 (0.39%)	ko00720
	Sulfur metabolism	22 (0.32%)	ko00920
Xenobiotics biodegradation and metabolism	Aminobenzoate degradation	71 (1.02%)	ko00627
	Chloroalkane and chloroalkene degradation	56 (0.81%)	ko00625
	Bisphenol degradation	50 (0.72%)	ko00363
	Metabolism of xenobiotics by cytochrome P450	44 (0.63%)	ko00980
	Drug metabolism - cytochrome P450	39 (0.56%)	ko00982
	Benzoate degradation	33 (0.48%)	ko00362
	Naphthalene degradation	27 (0.39%)	ko00626
	Caprolactam degradation	23 (0.33%)	ko00930
	Polycyclic aromatic hydrocarbon degradation	22 (0.32%)	ko00624
	Drug metabolism - other enzymes	15 (0.22%)	ko00983
	Ethylbenzene degradation	12 (0.17%)	ko00642
	Styrene degradation	9 (0.13%)	ko00643
	Dioxin degradation	6 (0.09%)	ko00621
	Chlorocyclohexane and chlorobenzene degradation	5 (0.07%)	ko00361
	Toluene degradation	3 (0.04%)	ko00623
	DDT degradation	1 (0.01%)	ko00351
	Fluorobenzoate degradation	1 (0.01%)	ko00364
Metabolism of cofactors and vitamins	Pantothenate and CoA biosynthesis	68 (0.98%)	ko00770

	Porphyrin and chlorophyll metabolism	35 (0.5%)	ko00860
	One carbon pool by folate	32 (0.46%)	ko00670
	Folate biosynthesis	24 (0.35%)	ko00790
	Riboflavin metabolism	21 (0.3%)	ko00740
	Nicotinate and nicotinamide metabolism	13 (0.19%)	ko00760
	Ubiquinone and other terpenoid-quinone biosynthesis	10 (0.14%)	ko00130
	Retinol metabolism	9 (0.13%)	ko00830
	Biotin metabolism	8 (0.12%)	ko00780
	Vitamin B6 metabolism	7 (0.1%)	ko00750
	Thiamine metabolism	3 (0.04%)	ko00730
	Lipoic acid metabolism	2 (0.03%)	ko00785
Metabolism of other amino acids	beta-Alanine metabolism	55 (0.79%)	ko00410
	Glutathione metabolism	53 (0.76%)	ko00480
	Selenocompound metabolism	18 (0.26%)	ko00450
	Cyanoamino acid metabolism	11 (0.16%)	ko00460
	Taurine and hypotaurine metabolism	8 (0.12%)	ko00430
	Phosphonate and phosphinate metabolism	7 (0.1%)	ko00440
	D-Arginine and D-ornithine metabolism	3 (0.04%)	ko00472
Metabolism of terpenoids and polyketides	Limonene and pinene degradation	38 (0.55%)	ko00903
	Terpenoid backbone biosynthesis	30 (0.43%)	ko00900
	Geraniol degradation	28 (0.4%)	ko00281
	Polyketide sugar unit biosynthesis	6 (0.09%)	ko00523
	Biosynthesis of vancomycin group antibiotics	5 (0.07%)	ko01055
	Sesquiterpenoid and triterpenoid biosynthesis	2 (0.03%)	ko00909
	Carotenoid biosynthesis	2 (0.03%)	ko00906

	Zeatin biosynthesis	1 (0.01%)	ko00908
	Biosynthesis of ansamycins	1 (0.01%)	ko01051
Glycan biosynthesis and metabolism	N-Glycan biosynthesis	37 (0.53%)	ko00510
	Glycosylphosphatidylinositol(GPI)-anchor biosynthesis	32 (0.46%)	ko00563
	Various types of N-glycan biosynthesis	30 (0.43%)	ko00513
	Glycosaminoglycan degradation	10 (0.14%)	ko00531
	Other glycan degradation	8 (0.12%)	ko00511
	Other types of O-glycan biosynthesis	7 (0.1%)	ko00514
	Glycosphingolipid biosynthesis - globo series	3 (0.04%)	ko00603
	Glycosphingolipid biosynthesis - ganglio series	2 (0.03%)	ko00604
Biosynthesis of other secondary metabolites	Tropane, piperidine and pyridine alkaloid biosynthesis	20 (0.29%)	ko00960
	Streptomycin biosynthesis	13 (0.19%)	ko00521
	Isoquinoline alkaloid biosynthesis	11 (0.16%)	ko00950
	Penicillin and cephalosporin biosynthesis	11 (0.16%)	ko00311
	Phenylpropanoid biosynthesis	7 (0.1%)	ko00940
	Stilbenoid, diarylheptanoid and gingerol biosynthesis	7 (0.1%)	ko00945
	Novobiocin biosynthesis	5 (0.07%)	ko00401
	Betalain biosynthesis	3 (0.04%)	ko00965
	Indole alkaloid biosynthesis	3 (0.04%)	ko00901
	Butirosin and neomycin biosynthesis	1 (0.01%)	ko00524
	beta-Lactam resistance	1 (0.01%)	ko00312

Genetic Information Processing	Translation	RNA transport	478 (6.89%)	ko03013
		Ribosome biogenesis in eukaryotes	226 (3.26%)	ko03008
		mRNA surveillance pathway	153 (2.2%)	ko03015
		Ribosome	131 (1.89%)	ko03010
		Aminoacyl-tRNA biosynthesis	61 (0.88%)	ko00970
	Transcription	Spliceosome	229 (3.3%)	ko03040
		Basal transcription factors	86 (1.24%)	ko03022
		RNA polymerase	57 (0.82%)	ko03020
	Folding, sorting and degradation	Protein processing in endoplasmic reticulum	213 (3.07%)	ko04141
		RNA degradation	175 (2.52%)	ko03018
		Ubiquitin mediated proteolysis	150 (2.16%)	ko04120
		Proteasome	43 (0.62%)	ko03050
		SNARE interactions in vesicular transport	32 (0.46%)	ko04130
		Protein export	28 (0.4%)	ko03060
		Sulfur relay system	17 (0.24%)	ko04122
	Replication and repair	Nucleotide excision repair	104 (1.5%)	ko03420
		DNA replication	82 (1.18%)	ko03030
		Mismatch repair	73 (1.05%)	ko03430
		Base excision repair	59 (0.85%)	ko03410
		Fanconi anemia pathway	51 (0.73%)	ko03460
		Homologous recombination	46 (0.66%)	ko03440
		Non-homologous end-joining	31 (0.45%)	ko03450
Environmental	Signal transduction	MAPK signaling pathway - yeast	445 (6.41%)	ko04011

Information Processing				
		MAPK signaling pathway	76 (1.09%)	ko04010
		Phosphatidylinositol signaling system	70 (1.01%)	ko04070
		Calcium signaling pathway	62 (0.89%)	ko04020
		Wnt signaling pathway	60 (0.86%)	ko04310
		Two-component system	55 (0.79%)	ko02020
		mTOR signaling pathway	43 (0.62%)	ko04150
		ErbB signaling pathway	40 (0.58%)	ko04012
		VEGF signaling pathway	38 (0.55%)	ko04370
		Jak-STAT signaling pathway	22 (0.32%)	ko04630
		Hedgehog signaling pathway	17 (0.24%)	ko04340
		TGF-beta signaling pathway	15 (0.22%)	ko04350
		Notch signaling pathway	13 (0.19%)	ko04330
		NF-kappa B signaling pathway	9 (0.13%)	ko04064
		MAPK signaling pathway - fly	4 (0.06%)	ko04013
	Membrane transport	ABC transporters	62 (0.89%)	ko02010
		Bacterial secretion system	6 (0.09%)	ko03070
	Signaling molecules and interaction	Neuroactive ligand-receptor interaction	8 (0.12%)	ko04080
Human Diseases	Infectious diseases: Bacterial	Pathogenic Escherichia coli infection	369 (5.32%)	ko05130
		Tuberculosis	68 (0.98%)	ko05152
		Salmonella infection	66 (0.95%)	ko05132
		Vibrio cholerae infection	50 (0.72%)	ko05110
		Shigellosis	45 (0.65%)	ko05131
		Bacterial invasion of epithelial cells	39 (0.56%)	ko05100

	Legionellosis	38 (0.55%)	ko05134
	Epithelial cell signaling in Helicobacter pylori infection	34 (0.49%)	ko05120
	Pertussis	19 (0.27%)	ko05133
Cardiovascular diseases	Viral myocarditis	172 (2.48%)	ko05416
	Dilated cardiomyopathy	23 (0.33%)	ko05414
	Arrhythmogenic right ventricular cardiomyopathy (ARVC)	9 (0.13%)	ko05412
	Hypertrophic cardiomyopathy (HCM)	9 (0.13%)	ko05410
Infectious diseases: Viral	Epstein-Barr virus infection	169 (2.43%)	ko05169
	HTLV-I infection	167 (2.41%)	ko05166
	Influenza A	110 (1.58%)	ko05164
	Herpes simplex infection	76 (1.09%)	ko05168
	Hepatitis C	40 (0.58%)	ko05160
	Measles	37 (0.53%)	ko05162
Cancers: Overview	Pathways in cancer	168 (2.42%)	ko05200
	Transcriptional misregulation in cancer	38 (0.55%)	ko05202
Neurodegenerative diseases	Huntington's disease	140 (2.02%)	ko05016
	Alzheimer's disease	91 (1.31%)	ko05010
	Parkinson's disease	58 (0.84%)	ko05012
	Prion diseases	42 (0.61%)	ko05020
	Amyotrophic lateral sclerosis (ALS)	22 (0.32%)	ko05014
Cancers: Specific types	Thyroid cancer	92 (1.33%)	ko05216
	Glioma	36 (0.52%)	ko05214
	Colorectal cancer	29 (0.42%)	ko05210
	Prostate cancer	28 (0.4%)	ko05215

	Renal cell carcinoma	24 (0.35%)	ko05211
	Acute myeloid leukemia	18 (0.26%)	ko05221
	Pancreatic cancer	18 (0.26%)	ko05212
	Non-small cell lung cancer	16 (0.23%)	ko05223
	Endometrial cancer	13 (0.19%)	ko05213
	Bladder cancer	11 (0.16%)	ko05219
	Chronic myeloid leukemia	8 (0.12%)	ko05220
	Melanoma	5 (0.07%)	ko05218
	Small cell lung cancer	3 (0.04%)	ko05222
	Basal cell carcinoma	1 (0.01%)	ko05217
Substance dependence	Alcoholism	67 (0.97%)	ko05034
	Amphetamine addiction	49 (0.71%)	ko05031
	Morphine addiction	34 (0.49%)	ko05032
	Cocaine addiction	20 (0.29%)	ko05030
	Nicotine addiction	4 (0.06%)	ko05033
Infectious diseases: Parasitic	Amoebiasis	26 (0.37%)	ko05146
	Toxoplasmosis	16 (0.23%)	ko05145
	Chagas disease (American trypanosomiasis)	11 (0.16%)	ko05142
	Leishmaniasis	5 (0.07%)	ko05140
Immune diseases	Systemic lupus erythematosus	26 (0.37%)	ko05322
	Rheumatoid arthritis	16 (0.23%)	ko05323
	Primary immunodeficiency	4 (0.06%)	ko05340
Endocrine and metabolic diseases	Type II diabetes mellitus	23 (0.33%)	ko04930
	African trypanosomiasis	10 (0.14%)	ko05143
	Type I diabetes mellitus	4 (0.06%)	ko04940

Cellular Processes	Cell growth and death	Cell cycle - yeast	321 (4.62%)	ko04111
		Meiosis - yeast	213 (3.07%)	ko04113
		Cell cycle	143 (2.06%)	ko04110
		Oocyte meiosis	110 (1.58%)	ko04114
		p53 signaling pathway	41 (0.59%)	ko04115
		Apoptosis	38 (0.55%)	ko04210
		Cell cycle - Caulobacter	4 (0.06%)	ko04112
	Transport and catabolism	Endocytosis	146 (2.1%)	ko04144
		Peroxisome	115 (1.66%)	ko04146
		Phagosome	106 (1.53%)	ko04145
		Lysosome	62 (0.89%)	ko04142
		Regulation of autophagy	27 (0.39%)	ko04140
	Cell communication	Tight junction	132 (1.9%)	ko04530
		Focal adhesion	49 (0.71%)	ko04510
		Gap junction	38 (0.55%)	ko04540
		Adherens junction	36 (0.52%)	ko04520
	Cell motility	Regulation of actin cytoskeleton	95 (1.37%)	ko04810
Organismal Systems	Immune system	Fc gamma R-mediated phagocytosis	84 (1.21%)	ko04666
		Chemokine signaling pathway	56 (0.81%)	ko04062
		Natural killer cell mediated cytotoxicity	39 (0.56%)	ko04650
		T cell receptor signaling pathway	29 (0.42%)	ko04660
		Cytosolic DNA-sensing pathway	24 (0.35%)	ko04623
		Leukocyte transendothelial migration	24 (0.35%)	ko04670
		Antigen processing and presentation	22 (0.32%)	ko04612

	B cell receptor signaling pathway	19 (0.27%)	ko04662
	RIG-I-like receptor signaling pathway	16 (0.23%)	ko04622
	NOD-like receptor signaling pathway	10 (0.14%)	ko04621
	Fc epsilon RI signaling pathway	9 (0.13%)	ko04664
	Toll-like receptor signaling pathway	9 (0.13%)	ko04620
Endocrine system	Insulin signaling pathway	72 (1.04%)	ko04910
	PPAR signaling pathway	51 (0.73%)	ko03320
	Progesterone-mediated oocyte maturation	45 (0.65%)	ko04914
	Adipocytokine signaling pathway	43 (0.62%)	ko04920
	GnRH signaling pathway	40 (0.58%)	ko04912
	Melanogenesis	34 (0.49%)	ko04916
	Renin-angiotensin system	8 (0.12%)	ko04614
Excretory system	Vasopressin-regulated water reabsorption	68 (0.98%)	ko04962
	Endocrine and other factor-regulated calcium reabsorption	37 (0.53%)	ko04961
	Aldosterone-regulated sodium reabsorption	17 (0.24%)	ko04960
	Collecting duct acid secretion	14 (0.2%)	ko04966
	Proximal tubule bicarbonate reclamation	8 (0.12%)	ko04964
Digestive system	Bile secretion	63 (0.91%)	ko04976
	Pancreatic secretion	31 (0.45%)	ko04972
	Salivary secretion	30 (0.43%)	ko04970
	Gastric acid secretion	30 (0.43%)	ko04971
	Mineral absorption	22 (0.32%)	ko04978
	Protein digestion and absorption	11 (0.16%)	ko04974
	Fat digestion and absorption	9 (0.13%)	ko04975
	Carbohydrate digestion and absorption	2 (0.03%)	ko04973

	Vitamin digestion and absorption	1 (0.01%)	ko04977
Nervous system	Dopaminergic synapse	60 (0.86%)	ko04728
	Glutamatergic synapse	51 (0.73%)	ko04724
	GABAergic synapse	49 (0.71%)	ko04727
	Long-term potentiation	47 (0.68%)	ko04720
	Synaptic vesicle cycle	43 (0.62%)	ko04721
	Serotonergic synapse	35 (0.5%)	ko04726
	Retrograde endocannabinoid signaling	34 (0.49%)	ko04723
	Neurotrophin signaling pathway	31 (0.45%)	ko04722
	Cholinergic synapse	29 (0.42%)	ko04725
	Long-term depression	17 (0.24%)	ko04730
Development	Axon guidance	38 (0.55%)	ko04360
	Osteoclast differentiation	20 (0.29%)	ko04380
	Dorso-ventral axis formation	4 (0.06%)	ko04320
Circulatory system	Vascular smooth muscle contraction	37 (0.53%)	ko04270
	Cardiac muscle contraction	10 (0.14%)	ko04260
Sensory system	Phototransduction - fly	26 (0.37%)	ko04745
	Olfactory transduction	23 (0.33%)	ko04740
	Taste transduction	19 (0.27%)	ko04742
	Phototransduction	14 (0.2%)	ko04744
Environmental adaptation	Plant-pathogen interaction	20 (0.29%)	ko04626
	Circadian rhythm - mammal	9 (0.13%)	ko04710
	Circadian rhythm - plant	4 (0.06%)	ko04712
	Circadian rhythm - fly	1 (0.01%)	ko04711
